# The Interdisciplinary eHealth Team: Chronic Care for the Future

**DOI:** 10.2196/jmir.6.3.e22

**Published:** 2004-09-03

**Authors:** John Wiecha, Timothy Pollard

**Affiliations:** ^1^Distance Education for HealthDepartment of Family MedicineBoston University Medical CampusBoston MAUSA

**Keywords:** Patient care team, medical informatics, telemedicine, interdisciplinary communication, quality of health care, chronic disease

## Abstract

An interdisciplinary clinical team is a consistent grouping of people from relevant clinical disciplines, ideally inclusive of the patient, whose interactions are guided by specific team functions and processes to achieve team-defined favorable patient outcomes. Teamwork supported by properly designed eHealth applications could help create more effective systems of care for chronic disease. Given its synchronous and asynchronous communication capacity and information-gathering and -sharing capabilities, the Internet is a logical platform for supporting interdisciplinary clinical teamwork. Research is needed to better understand how interdisciplinary eHealth team members can work together in everyday practice and to guide the development of effective and efficient eHealth software applications to support greater clinical teamwork.

## Introduction

Chronic diseases are the most common cause of mortality and morbidity in developed countries [1]. The increasing complexity of chronic disease managed in the ambulatory setting, and the expanding evidence base available to guide medical care, has led to calls for interdisciplinary team models of patient management [2] that include the patient [3].

The effective and efficient functioning of health care teams is predicated on two factors amenable to information technology solutions: patient data and a workable method of coordinating interactions among team members.

Teamwork supported by properly designed eHealth applications could help create more effective systems of care for chronic disease. However, new eHealth models have not emerged to reach this potential, nor has there been general recognition of the contribution that electronic technology can make to promoting clinical teamwork or the need to rigorously evaluate the facilitation of clinical teamwork via electronic means.

### Team Theory

Although teamwork in health care has been promoted as beneficial since the turn of the century [4], consensus on a definition of clinical teamwork is not apparent in the literature.

Lorimer et al [5] suggest that "a team is a small number of consistent people committed to a relevant shared purpose, with common performance goals, complementary and overlapping skills, and a common approach to their work. Team members hold themselves mutually accountable, team results are outcomes."

This definition implies *interdisciplinary* interactions, in which all members participate in the team's activities and rely on one another to accomplish goals. In contrast, in the "multidisciplinary" team model, health care providers tend to treat patients independently and to share information with each other, while the patient may be a mere recipient of care. An interdisciplinary team aspires to a more profound level of collaboration, in which constituents of different backgrounds combining their knowledge mutually complete different levels of planned care [4].

There is growing advocacy for including patients as members of the teams that manage their chronic illness [6]. The burden of chronic illness is borne most heavily by patients and their families, as most care of chronically ill patients takes place in the home [7]. The U.S. Institute of Medicine [2] as well as other authorities have argued that for successful treatment of chronic illness, patients must be "well informed about their disease, know where they can access treatment, and have greater control over their treatment" [8]. There is good evidence that patients should be "partners in their care" [8]. Integrating patients into the virtual health care team is an obvious next step in this evolution.

### eHealth Applications for Teamwork

Given its synchronous and asynchronous communication capacity and information-gathering and -sharing capabilities, the Internet is a logical platform for supporting interdisciplinary teamwork. This concept is not new. As early as 1968, two of the founders of the modern-day Internet wrote:

We believe that communicators have to do something nontrivial with the information they send and receive. And we believe that we are entering a technological age in which we will be able to interact with the richness of living information―not merely in the passive way that we have become accustomed to using books and libraries, but as active participants in an ongoing process, bringing something to it through our interaction with it, and not simply receiving from it by our connection to it. . . . We want to emphasize something beyond its one-way transfer: the increasing significance of the jointly constructive, the mutually reinforcing aspect of communication― the part that transcends 'now we both know a fact that only one of us knew before.' When minds interact, new ideas emerge [9].

Defining how minds actually interact in a clinical team, and having a clear understanding of team structure and function, is essential to building successful interdisciplinary care teams that function electronically.

 Four domains of team function have been described [10] that can guide interdisciplinary eHealth team development, evaluation, and research. These include structure (composition and representation), context (relationship to the larger institution), process (of team functioning), and productivity (measure of impact). The structure of teams refers to the membership composition and their hierarchic organization. The context is shaped by environmental structure and financial and organizational relationships. Team process is determined by which methods are used for team communication, by the hierarchic nature of the team, by the values of team members concerning power sharing, and by idiosyncratic relationships that develop within teams. Productivity can be understood in the same way as individual productivity. Of these four domains, the *process* of eHealth teams is likely to most differ from teams supported by non-electronic means of communication and information sharing, and so a deeper understanding of process is in order. Team process, based on the work of Heinemann [10] and others, can be characterized into what we call the "12 C's of teamwork":

***The 12 C's Defining Teamwork:***

1. Communication (this is the sine qua non of teamwork)
2. Cooperation (empowerment of team members)
3. Cohesiveness (team sticks together)
4. Commitment (investing in team process)
5. Collaboration (equality in the team)
6. Confronts problems directly
7. Coordination of efforts (insuring actions support a common plan)
8. Conflict management
9. Consensus decision making
10. Caring(patient centered outcomes)
11. Consistency (with one another and the environment)
12. Contribution (feeling this is being made)

Applying these 12 processes to a group might reasonably be expected to produce creative synergies among group members, producing new and perhaps unexpected ideas and solutions and resulting in a functional team. Diverse perspectives may contribute to creativity and learning, skill acquisition, and innovation.

In summary, a modern interdisciplinary team is a consistent grouping of people from relevant clinical disciplines, ideally inclusive of the patient, who interact guided by these 12 processes to achieve team-defined favorable patient outcomes.

### Evidence for Effectiveness of Teamwork in Clinical Settings 

The purported benefits of teamwork in health care are many, and include increased learning and development of people and organizations; better utilization of resources and planning for the future, ensuring the best use of resources and minimization of unnecessary costs; and improving job performance and work quality [11]. However, despite calls for reengineering health care processes to include greater teamwork, published studies on the effectiveness of teamwork provide conflicting results, and the state of research on teamwork has been rated poor [12].

In a 1999 review article, Schofield and Amodeo [13] analyzed research evaluating the impact of clinical teamwork. They reported significant weaknesses in research rigor, with great inconsistency in terminology and little empirical evidence for the efficacy of interdisciplinary teams at that time.

More recently, there has emerged some research evidence demonstrating teamwork benefit [10,14-18]. For example, Gittell et al [17] studied the effect of several key dimensions of coordination, including communication, shared goals, shared knowledge, problem solving, and mutual respect, on the quality of orthopedic surgical care. The more coordination the team demonstrated, the better the patients' postoperative functioning and the shorter the hospital stays.

Teamwork under the guise of "collaborative care" or "shared care" schemes has been described and evaluated and has improved patient outcomes [19].

Historically, psychiatric disorders have been managed by either psychiatrists, psychologists, or primary care physicians. Care models that include patient education, psychiatric and primary care co-management of drugs, and case management have been shown to improve patient outcomes 20]. Patients with depression rated the quality of their care more highly [21,22], were more adherent to medications [20,23], had fewer symptomatic days [24,25], and decreased depression scores [22,26] when treated collaboratively. Although the cost of care was higher in these models due mostly to increased patient compliance with visits and medicines [27,28], these costs were offset at the societal level by increased days of work [25]. Similar results have been reported with panic disorder [29]. How these collaborative models improve outcomes is not clear.

### Limitations of Research on eHealth Teamwork

Although there is some evidence demonstrating improved clinical outcomes by virtue of good team performance, there has been little work on the relationship between team process and clinical outcomes [10]. In other words, we do not know why teamwork improves clinical outcomes, and therefore we do not know which processes ought to be electronically enhanced.

In fact, many of the assertions regarding effective attributes of a successful team do not have supporting evidence. Various attributes have been promoted as the essential qualities of a successful interdisciplinary team, including diversity of participants;

shared records; improved communication between doctors and patients; a clear role for the patient; specialist input; consensus on management; and close coordination [14]. It has also been argued that diversity of professional, cultural, and demographic characteristics provides varied perspectives on decision making and may improve problem solving and creativity [10].

New methods are needed to evaluate health care teams. Although there is a substantial body of literature on teamwork, methodological weaknesses are prevalent. Use of non-validated instruments, poorly defined methods and measures, lack of control groups, and inadequate isolation of specific teamwork effects upon outcomes contribute to our ignorance. There is little research at the clinical trial level evaluating various methods of online or conventional clinical teamwork, and there is limited research on interdisciplinary teamwork in community-based primary care settings. Most studies offer only "explanatory hypotheses or sociological theories" [30].

Although strong provider-patient relationships can positively influence patient satisfaction, adherence to treatment, and health care outcomes [31], few studies have addressed how to meaningfully integrate the patient into a more broadly constituted interdisciplinary clinical team, virtual or otherwise. There is considerable discussion in the literature on how to set up teams and manage them, but research explaining how interdisciplinary team members manage their concerns and work together in everyday practice is minimal [12]. Likewise**,** we know little about models and effectiveness of electronically supported team interactions. New communications processes augmented by advances in electronic technology provide fertile soil for further research.

McCallin [12] and Schofield [13] have called for more sophisticated research on conventional and electronically mediated teamwork, making such points as: (1) Articles need to be more analytic and meet a higher standard of conceptualization; (2) all variables need to be specified, and a more sophisticated research design used when possible; (3) comparison groups, almost entirely absent from the current literature, should be used; (4) researchers should compare interdisciplinary team interventions with one-on-one interventions; and (5) more research is needed to understand the processes used by clinical team members as they work [12].

We agree that "there is an urgent need for more research into patients' information needs and preferences and for the development and evaluation of decision support mechanisms to enable patients to become informed participants in treatment decisions" [6]. This work should include research and development of eHealth applications focused on how these goals can be met within a broader context of collaboration among health care professionals caring for the patient.

A new system for asthma care provides an opportunity for research into the impact of electronic teams on patient care. A Web-based tool has recently been introduced in Germany (Forum-Telemedizin, or FTM) [32] to promote the self-management behaviors of children with asthma. FTM as currently implemented uses disease-specific data acquisition in the patients' home, educational tools that include Web-based learning games, point-of-care tools for physicians and nurses, and computer-driven adaptation to individual patient treatment and assessment needs. It is designed to improve patient motivation and self-care in youths most severely affected by asthma in clinical practice.

FTM will be modified by the authors to support data-driven teamwork among all health care professionals responsible for the care of the child with asthma, including primary care physicians, asthma specialists, asthma nurses, and school nurses. The system will be transparent, in that child and parent will be encouraged to be bonafide participants in the management discussions. A randomized clinical trial in progress should help to answer questions about which aspects of these systems are producing positive clinical outcomes, including the relative impact of telemonitoring with electromedical devices, direct contact with the physician, co-management via online teams, patient education, or combinations of the above. Studies are also needed to assess the impact of such systems on adult patients with chronic illness, as well as for the prevention of illness via promotion of healthy lifestyles.

### Conclusions

A recent review [33] noted the need for additional study of telemedicine in chronic conditions, with an emphasis on patient-centered approaches to care. The discourse ontelemedicine applications to date has not embraced the utility of telemedicine systems to promote clinical teamwork.

In the near future, we anticipate, the Internet and appropriately designed multifunctional software applications will enable teamwork to occur anywhere, at any time. The team could have access to real-time patient data sent from the home, and the patient could be fully integrated into a collaborative care process by accessing appropriate patient data and participating in communications between caregivers via asynchronous discussion threads. Ultimately, digital audio and video accessed over the Internet will be widely used to facilitate these communication processes.

A research agenda on the impact of eHealth applications should integrate investigations of clinical teamwork functionality. As we develop, implement, and evaluate new tools for integrated communication, remote patient education, and monitoring of patients with chronic diseases, we should be sure that facilitation and assessment of online clinical teamwork is an explicit functional goal. The current undeveloped state of research on the effectiveness and efficiency of clinical teamwork can be advanced by evaluating teamwork schemes that are facilitated electronically.

The research agenda should include development of models to guide the process by which effective and efficient teamwork can be promoted and supported online. Methods will need to be developed to measure the quantity and quality of online teamwork. A unique opportunity exists to assess the content of team interactions given the retrievable nature of online communication.

These records can provide a rich resource documenting teamwork characteristics and will be available for qualitative analyses, doing much to penetrate the "black box" of shared care. In the past, this work has been hampered by the lack of such enduring records and the impracticality of impartial observers accompanying health care providers to record team interaction.

To quote the president of the Association of American Medical Colleges when advocating rapid introduction of information technology into medicine and noting potential pitfalls, "One pitfall would be to embrace the technology, but to stop short of taking full advantage of its transforming potential" [34].
